# A Research Agenda for Malaria Eradication: Vector Control

**DOI:** 10.1371/journal.pmed.1000401

**Published:** 2011-01-25

**Authors:** The malERA Consultative Group on Vector Control

## Abstract

Different challenges are presented by the variety of malaria transmission environments present in the world today. In each setting, improved control for reduction of morbidity is a necessary first step towards the long-range goal of malaria eradication and a priority for regions where the disease burden is high. For many geographic areas where transmission rates are low to moderate, sustained and well-managed application of currently available tools may be sufficient to achieve local elimination. The research needs for these areas will be to sustain and perhaps improve the effectiveness of currently available tools. For other low-to-moderate transmission regions, notably areas where the vectors exhibit behaviours such as outdoor feeding and resting that are not well targeted by current strategies, new interventions that target predictable features of the biology/ecologies of the local vectors will be required. To achieve elimination in areas where high levels of transmission are sustained by very efficient vector species, radically new interventions that significantly reduce the vectorial capacity of wild populations will be needed. Ideally, such interventions should be implemented with a one-time application with a long-lasting impact, such as genetic modification of the vectorial capacity of the wild vector population.

## Abstract 2

The Malaria Eradication Research Agenda (malERA) Consultative Group on Vector Control outline the research needed to ensure vector control at every stage of malaria eradication.

**Summary Points**

- Improved vector control is essential for the elimination/eradication of malaria
- In regions where transmission rates are low or moderate, existing tools may be sufficient to achieve elimination but in many malaria-endemic regions, new vector control interventions, including new insecticides and formulations, are needed
- Better understanding of vector biology is an essential prerequisite for the development of new control interventions
- Sustained commitment to the development of radically new approaches such as the genetic modification of mosquitoes is critical to reduce the high vectorial capacity in some malaria-endemic regions
- Innovative cross-disciplinary technologies are needed to control outdoor biting and resting mosquito vectors, to measure transmission, and to educate communities about vector control

## Introduction

The overarching goal of malaria vector control is to reduce the vectorial capacity of local vector populations below the critical threshold needed to achieve a malaria reproduction rate (*R*_0_, the expected number of human cases that arise from each human case in a population) of less than 1. Because of the long extrinsic incubation time of *Plasmodium* in its *Anopheles* vectors, the most effective vector control strategies in use today rely on insecticide interventions like indoor residual insecticide sprays (IRSs) and long-lasting insecticide-treated nets (LLINs) that reduce vector daily survival rates [1]. For many malaria-endemic regions, these tools can make substantial contributions to malaria control and may be sufficient for local malaria elimination. These were the only regions considered by the recent Malaria Elimination Group (MEG). Regions where existing interventions will not be sufficiently effective include those where high rates of transmission occur. For example, in much of sub-Saharan Africa, where the entomological inoculation rates (EIRs) can reach levels approaching 1,000 infective bites per person per year [2],[3], the best use of existing interventions can only help to reduce annual inoculation rates by approximately an order of magnitude. Additional interventions will clearly be required, however, both for regions with extremely high rates of transmission and for regions where the major vectors are not susceptible to current control tools [4].

To develop vector-targeted interventions in support of malaria eradication in all disease endemic settings that are unfettered by these limitations, three challenges need to be recognized and addressed with great urgency today. The first challenge, for which near-term product development is essential, is the preservation and improvement of the utility of existing insecticide-based interventions. This challenge will require a vibrant research agenda that develops a broader range of insecticides with novel modes of action that can circumvent emerging resistance to existing insecticides, particularly the pyrethroids. This agenda must include the creation of strategies for the use of new insecticides that minimize the emergence of resistance. A related and critical focus of the agenda will be the development of rapid and affordable methods for detecting the emergence of epidemiologically important levels of insecticide resistance. Because of the fundamental dependence of many current malaria control and elimination programs on pyrethroid insecticide–based LLINs and the emerging problem of pyrethroid insecticide resistance in many vector species, especially in sub-Saharan Africa, development of new insecticides that can be used in LLINs is the most immediate need [5].

The second challenge is development of interventions that affect vector species not effectively targeted by current tools. At least three dozen different species of *Anopheles* mosquitoes are important in malaria transmission worldwide. Many of these species are not susceptible to tools like IRS and LLINs, which target indoor feeding and/or resting vectors [6]. Control of malaria transmitted by these vectors will require new interventions that target other aspects of their biology, including outdoor feeding and resting, oviposition site preference, mating behaviour, or sugar meal selection. Major features of the agenda to tackle this challenge will be defining the vector species for which such new tools are most important and devising tools that will be effective for multiple important vector species.

The most difficult research challenge for vector control during all phases of malaria elimination/eradication but particularly during the final stages of eradication is development of novel approaches that will permanently reduce the very high vectorial capacities of the dominant malaria vectors in sub-Saharan Africa. Without such approaches, local elimination in Africa will be extremely challenging. Even when elimination is achieved, the residual vectorial capacities of local mosquitoes will pose a lingering threat of massive epidemics should malaria be reintroduced to a population that has lost partial immunity. Measures to reduce vectorial capacities will need to be either extremely cost-effective, if they are to be sustained until eradication is achieved, or able to effectively yield a long-term, sustained reduction of transmission following a one-time application. Genetic control programs (which could be achieved by a variety of genetic manipulation approaches) designed to permanently reduce the vectorial capacities of natural vector populations have received the most attention to date, and currently represent some of the most promising ideas in this area [7], but the development of other, novel approaches must be strongly encouraged.

It is these three challenges that the malERA Consultative Group on Vector Control concentrated on during its deliberations, the results of which are presented here.

## Current Tools and Resource Gaps

The key goal of the malERA Consultative Group on Vector Control was to help define the research and development agenda that will be required to sustain and improve the effectiveness of currently available tools like LLINs and IRS and to develop new vector-targeted tools that can be used to interrupt transmission in environments or at intensities that these existing tools cannot reach. It is clear that new technology will be required in very high transmission areas to reduce vectorial capacity and achieve even effective control, let alone elimination. The main aim of this paper is to define a research and development agenda that focuses on those new research questions and knowledge gaps that arise specifically in response to the call for malaria eradication, and that would not otherwise be at the top of the agenda (Table 1). It is particularly important to recognize that this operationally specified goal significantly limits the scope of research and development under consideration, and this document should not be the basis for all vector research related to malaria. Our article does, however, describe the challenges for vector control methodology in the elimination phase, for detecting and monitoring areas of persistent transmission, and for detecting and monitoring nonrandom transmission leading to outbreaks. We also discuss the requirements for rapid and urgent intervention when outbreaks occur (see also [8]).

**Table 1:** Vector control interventions required for sustained control and for eradication.

Sustained Control	Eradication
	Better vector monitoring and evaluation information to target interventions
Effective insecticides for LLINs and/or IRS	Effective insecticides for LLINs and/or IRS
Resistance monitoring and management	Resistance monitoring and management
Vector identification and incrimination	Vector identification and incrimination
Appropriate integrated vector management	Appropriate integrated vector management
	Targeted interventions for outdoor biting and resting mosquitoes
	Novel approaches to reduce permanently the high vectorial capacity of major vectors (e.g., genetic modification)
	Effective consumer products for vector control

The Consultative Group identified four key components to successful vector control within an eradication agenda. First, the ecology of vectors responsible for malaria transmission in those regions of the world where current tools are insufficient for elimination needs to be understood. Second, sets of synergistic or complementary interventions tools need to be developed and applied through rationally designed programs that can be spatially and temporally combined into effective intervention programs. Third, appropriate monitoring and evaluation tools that can guide the application and evolution of control and elimination programs as malaria endemicity is pushed towards local elimination need to be developed and applied. Finally, there is a critical need for built-in flexibility in programs so that where initial efforts fail, they can adapt to circumstances by incorporating and implementing new approaches. Thus, as malaria programs are scaled up, vector control will have a major role in disease burden reduction but, as programs become increasingly successful in reducing transmission, accurate estimation of the point at which large-scale vector control activities can be relaxed will become critical. Premature removal of mainstream vector control, either through planned reductions in activities or through failure of long-lasting interventions like LLINs or IRS as resistance evolves, is likely in many instances to lead to a catastrophic increase in morbidity and mortality because of resurgent malaria in a nonimmune population [8],[9].

The exact role of vector control as countries enter the elimination phase of activities will be situation specific. However, valuable lessons can be drawn from the WHO Global Malaria Eradication Program (GMEP) of the 1950s and 1960s [10], in which vector control alone was considered to be enough in many situations to eliminate malaria. Although this approach was successful in some cases, success was often short-lived [11],[12]. Another valuable lesson can be learned from current efforts to eradicate filariasis. For this vector-borne disease, multiple rounds of mass drug administration in many countries divorced from targeted vector control have not achieved the predicted interruption in transmission [13].

Indeed, there is now a consensus that malaria elimination with current tools is far more likely if the best available tools are used in combinations. In the past two decades, especially in an African context, the combination of drugs and vector control with impregnated nets has been highlighted for its role in the reduction of morbidity and mortality [14]. However as malERA sets out a research and development agenda for elimination/eradication and vector control, other interventions must be considered primarily in terms of their impact on malaria infection and transmission, not instead of, but in addition to, their role in prevention and modification of disease.

We highlight the research and development areas identified as priority areas by the Consultative Group before providing a summary research and development agenda that draws together the various strands of our discussions.

## The Development of a Formal Analytical Framework

**Figure 1:**
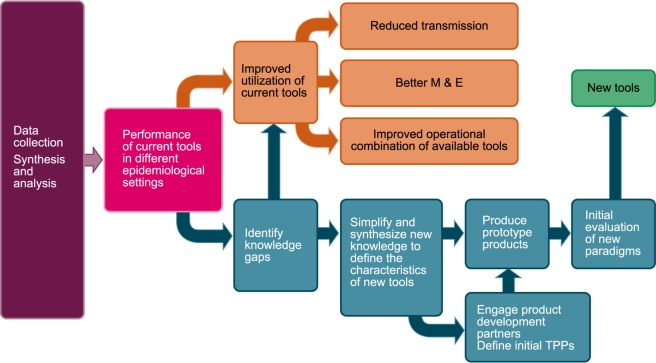
A formalized analytical framework for the collection, analysis, and central presentation of relevant information. M&E, monitoring and evaluation. Image credit: Fusión Creativa.

The malaria eradication agenda would clearly be advanced by the development of a formalized analytical framework that facilitates the collection, analysis, and central presentation of relevant information (Figure 1). Such a framework could significantly help elimination/eradication programs optimize the use of current vector control tools. In addition, when available tools are properly deployed and transmission persists, such a framework could also highlight the knowledge gaps that currently limit accurate development of clear target product profiles (TPPs) for new tools. The generation and sharing of information from systematic assessments of the results of malaria elimination programs across different epidemiological settings will help drive the development of new technologies that will be needed to achieve elimination in more intransigent transmission settings.

The most immediate task of the analytical framework will be to focus research and development resources on the malaria transmission settings for which new or improved elimination tool development is most critical. These settings include much of sub-Saharan Africa and parts of Papua New Guinea, regions where vector populations are capable of sustaining transmission at high vectorial capacities that significantly exceed the possibility of elimination with current tools. In addition, however, there may be other malaria transmission regions of more modest vectorial capacity where important current tools such as IRS and LLINs have little impact because the important vectors do not enter houses to rest or to seek blood meals. Some information already exists that can be brought together to define these high-risk regions [15]–[17]. For other regions, however, problems may become obvious only when the application of current interventions proves insufficient.

The analytical framework should systematically coordinate available data from disparate multidisciplinary resources, including both peer-reviewed and “grey” literature, via a Web portal to facilitate access and analysis. The Consultative Group's recommendation is that disparate multidisciplinary resources are brought together in a coherent format that will allow the objective assessment of the knowledge base as it relates to the performance of current tools. The ideal format would allow the systematic assessment of issues arising in countries that have already eliminated malaria and in countries that are still in the first wave of malaria elimination, in isolation and in combination, and would allow comparisons to be made of tool performance in different epidemiological settings. Some of this information—for example, the worldwide distribution of malaria risk and information on the worldwide distribution of important malaria vectors—already exists in centralized resources and needs only be made more readily available. However, other kinds of important information will need to be assembled from disparate sources (for example, field data from major malaria research and control programs and the very significant but inaccessible literature that emerged from the first GMEP) or generated de novo (for example, the determination of the specific malaria transmission behaviours of vectors that have only recently been determined to be members of cryptic species complexes [18]).

As the elimination agenda progresses, this growing body of information can be used to develop and use models of vector biology and transmission and to test intervention hypotheses such as the effect of combining available control tools into integrated control programs. Further, modeling can be used to identify opportunities to develop new interventions and establish the settings where vector control–targeted interventions are inappropriate. It will be particularly important to invest in new interventions that are likely to impact additively or even synergistically with existing tools. Modeling can be an important first step in evaluating such potential interactions (also see [9]).

## The Preservation and Improvement of Current Tools

The obvious major threat to current vector-targeted interventions is insecticide resistance, and addressing this problem will be both an important near-term research concern and a continuing, long-term concern as new insecticide formulations and ingredients are developed and used. Furthermore, this problem is critical for control efforts as well as for elimination and eradication efforts. Pyrethroids are the only insecticides currently used operationally on LLINs and are also the dominant insecticide class in IRS, but resistance to this insecticide class is now widespread, with multiple resistance mechanisms spreading in the two major African malaria vectors [19],[20]. Although the operational impact of these resistance indicators remains to be established, multiple studies have demonstrated the direct association of resistance measures with entomological indicators such as mosquito mortality, biting rates, and blood feeding success.

Sporadic insecticide resistance monitoring is undertaken by control programs, predominantly using WHO bioassays, but the results from these bioassays are rarely linked to any assessment of control failure. Moreover, resistance-monitoring efforts are not typically used to provide formal guidance to control programs on the selection of alternative vector control strategies in the presence of resistance. Because of the very large number of vector species, the many insecticides in use, and the large numbers of potential resistance mechanisms, choosing the correct vector control strategy is clearly a complex and daunting problem. An essential first step towards developing a rational solution will be to develop and provide new tools for the quantitative monitoring of different forms of resistance in different vector species. Monitoring could be done through the provision of public protocols, through training and the provision of kits, or by establishing a regional service. The potential complexity of meaningful data generation and interpretation suggests that the last option may be preferable. Indeed, we note that this type of activity could readily be combined in a monitoring and evaluation framework with a laboratory service that provides drug resistance or serology monitoring capability. In addition, data on the temporal and geographic distribution of insecticide resistance need to be efficiently assembled and made publicly available through a formal analytic framework to help guide both control program and research decisions.

LLINs, IRS, and larvicides attack different behaviours or life stages of the mosquito. There is some evidence that LLINs and IRS used in combination may be synergistic, although both target adult female mosquitoes indoors [21]. Within an eradication agenda, the cost-effectiveness and benefits of such combinations need to be assessed. The recommendation of the Consultative Group, therefore, is that potential combinations of present and new control tools be explored theoretically in a modeling framework [9], and that potentially optimal integrated vector management strategies be tested in large-scale field trials in different epidemiological settings to assess their ability to reduce transmission and the burden of disease. If insecticide resistance dramatically reduces our ability to reduce transmission, it becomes a major threat to eradication, and mitigating strategies must be tested in the field to contain resistance in the absence of new alternative insecticides. Finally, insecticide-resistance management technologies need to be developed for the future that use combinations of vector control tools that do not depend on the main classes of insecticide in current use. Such combinations might include repellents, larvicides, environmental management, and possibly pathogens.

## Improvement of the Knowledge Base of Vector Ecology

Malaria is transmitted in diverse epidemiological situations by a wide range of potential combinations of “primary” and “secondary” vectors. Moreover, most widely recognized vector species are members of cryptic species complexes [18] and even within currently recognized complexes, further heterogeneities may exist in vector population structure that can limit the effectiveness of control tools [22]. The present vector control tools (LLINs and IRS) were developed to reduce transmission in areas where the primary vectors feed and/or rest indoors. When these interventions are implemented under optimal programmatic conditions, diligent monitoring will identify areas where there are limitations in their effectiveness.

Failure to achieve the expected level of control may result from a number of factors. Complex mixtures of vector species may be present, including vectors with outdoor biting and resting behaviours, or a more complex genetic structure within recognized vector taxa. Moreover, vector populations can develop behavioural as well as physiological forms of resistance to insecticides. To assess the possible impact of behavioural evolution on the effectiveness of vector control tools, and to better target vector species or populations escaping these tools, we have to understand both larval and adult ecologies and behaviours. At the present, we have only a limited understanding of the ecology and population structure of some of the major vectors, such as *An. gambiae* in Africa. Unfortunately, even less is known about where many of the other important vectors feed, rest, mate, and oviposit, or about their population structure, or even the extent of their geographic distributions. These deficiencies are due to both the lack of adequate sampling and monitoring methods and a historical lack of emphasis on the study of population biology of malaria vectors in many parts of the world.

Development of TPPs for new interventions that could supplement existing control tools will require knowledge of the critical points in the biology of different vector species. These points should be features of a vector's biology that are sufficiently predictable to constitute a target for the control tool, such as a predictable resting, blood- or sugar-feeding, oviposition, or mating site. Technologies (see later) that enable accurate tracking of mosquito movement in space and time are needed to establish these critical points in the biology of different vector species.

## The Development of New Vector-Targeted Interventions

### Near-Term Translation of Appropriate Interventions

Malaria vector control activities today are heavily reliant on the distribution of LLINs or IRS. In some instances, these are augmented with larval control or fortuitously complemented by social housing schemes or economic development that negatively impact on *Anopheles* mosquito breeding. This limited armamentarium is, in part, the legacy of a malaria control approach developed before and during the GMEP of the 1950s and 1960s that was followed by a shift in the 1970s through the 1990s away from the interruption of transmission to the control of morbidity and mortality based largely on chemotherapy [11],[12]. Research on the control of malaria transmission was consequently very limited and poorly coordinated both during the time of the GMEP, which was characterized by overoptimistic expectations of the effectiveness of DDT, and in the years that followed when transmission was no longer the main concern. Nonetheless, a number of proposed alternative vector control methods have emerged recently, most of which have not yet been extensively evaluated and developed (see Table 2 for some examples). What is badly needed is a well-defined development pipeline to ensure that the more promising among these alternative methods are brought into mainstream operation and that the less appropriate are down-selected.

**Table 2:** Examples of novel tool development and intended objectives.

Sustained-Use Interventions		Time-Limited Interventions	
Category	Objective	Category	Objective
Insecticides and related chemical agents (synthetic and natural [“bio-prospecting”]) for environmental, dwelling, and systemic applications (humans or animals)	Control, elimination	Biological or chemical agents that affect age structure (decrease extrinsic incubation period, for example, *Wolbachia* spp.)	Control, elimination, eradication
House design to impede vector access and sustainability	Control, elimination	Genetic approaches to reduce adult longevity (“death-on infection” genes killing only those mosquitoes that become infected)	Control, elimination, eradication
Biological agents (plant, fungi, algae, predators, niche competitors, insect viruses, and other pathogens) for population suppression	Control, elimination	Biological agents targeting pathogens, for example, symbiotic organisms engineered to kill pathogens (paratransgenesis)	Control, elimination, eradication
Ecological/environmental modification (source reduction) targeting sites for breeding (oviposition), subadult development, and adult resting sites	Control, elimination	Genetic approaches targeting vector competence	Control, elimination, eradication
Chemical attractants/repellent agents (synthetic and natural [“bio-prospecting”]) for dwelling and personal applications that would target both indoor and outdoor biting	Control, elimination		

For example, the development of cost-effective longer lasting IRS formulations of different insecticide classes would remove the economic and logistical arguments that preclude the use of IRS in some settings. Today's heavy reliance on pyrethroids for both LLINs and IRS is driven both by a lack of new insecticides and by limited development in formulation technology. The latter problem is amenable to short-term resolution. Similarly, models suggest that interventions that act on older adult mosquitoes are less prone to resistance selection than traditional insecticides [23],[24], but this has still to be demonstrated operationally. Other insecticides have failed to cross the translational gap because the short residual shelf life of the formulations under operational conditions is a major barrier to their commercialization. Until this element of the critical pathway to commercial uptake is resolved, many promising insecticides are unlikely to play an active role in operational control.

**Figure 2:**
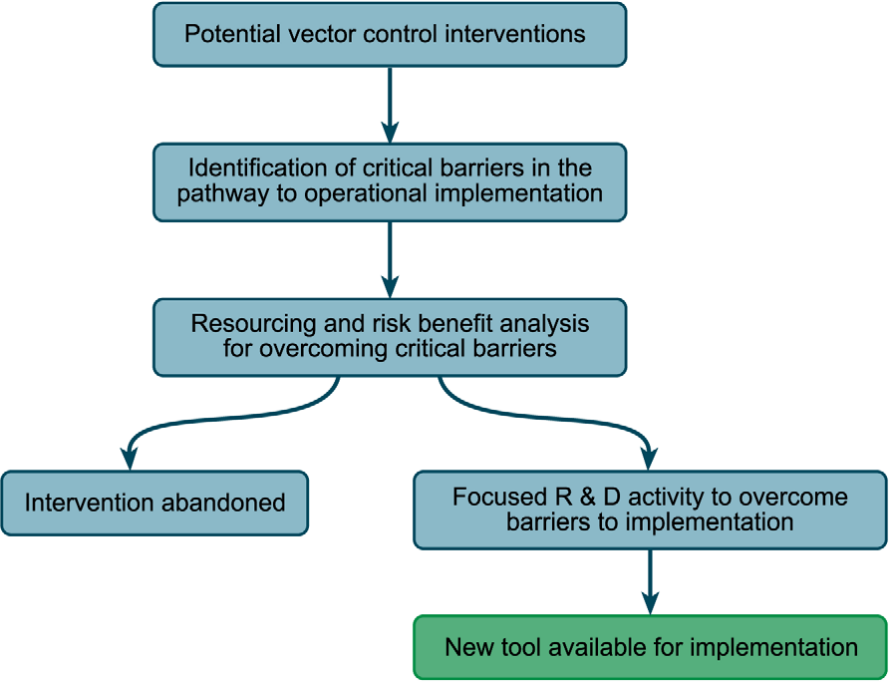
A scheme for the analysis of the development status of the different interventions; similar schemes are used in the commercial development of drugs, for example. Image credit: Fusión Creativa.

Novel tools need proper evaluation in field trials and, if their efficacy is demonstrated, they need testing in combination for their effect on infection and transmission. We recommend that in reviewing current and potential interventions within the analytical framework, a commercial-style analysis of the development status of the different interventions be undertaken (Figure 2) and the barriers on the critical pathway to implementation be identified. Once identified, the resources required to overcome these barriers can be established and an appropriate risk benefit analysis can be undertaken. This analysis will allow rapid movement away from long lists of potential vector control interventions and towards a better-defined list of actual interventions that can be coupled to clear guidance on appropriate deployment in the different stages of malaria elimination across a range of epidemiological settings. Analysis of the development status should also include modeling to guide selection and testing of combinations and settings where they should be introduced (see also [9]).

New vector control tools will be needed in the short and medium term as the current tools will be inadequate for malaria elimination in most settings. The strategy outlined above will allow researchers and developers to capitalize on information that is already in the public domain and to efficiently and cost-effectively develop the most appropriate new tools in the short term that could be useful additions to the armamentarium for malaria eradication.

### Longer-Term Development of Novel Sustained-Use Interventions

To achieve malaria eradication, we will need to reduce regional *R*_0_ to less than 1 and sustain transmission rates below this critical threshold until global eradication can be achieved. Achieving this goal will require both augmentation of current control methods and the development of novel interventions to interrupt transmission in ways that address a broad range of potential impediments, including political instability, geographic isolation, and the development of chemical or behavioural resistance. Perhaps the biggest difficulties will be the economic and social challenges that will be associated with the need to sustain the impact of such interventions in some regions for very long periods, possibly decades, until the risk of parasite reintroduction is no longer a concern.

Insecticide-based interventions for sustained use should not be compromised by resistance. Models of resistance management developed using data from control of agricultural insect pests and from other large-scale vector control efforts indicate that stable, long-term resistance management strategies require a minimum of three active ingredients. These ingredients need different modes of action, distinct metabolic detoxification pathways, and no resistance to any of them should be present at the onset of the program. The levels of resistance currently circulating in many mosquito vectors to all registered public health pesticides precludes such a system being established today [25]. Hence, it is vital that we continue to develop new active ingredients to replace existing insecticides when vectors develop significant resistance. The ideal goal would be to do this in a time frame that allows multiple new insecticides to be introduced together.

It is also important that a broader set of tools for targeting adult female vectors be developed so that adult vector survival rates and the resulting population age structure can be reduced to levels where insufficient older female mosquitoes capable of supporting parasite sporogonic development are present in the vector population. The most critical needs will be for vector control tools that complement existing methods by targeting aspects of the mosquito's life cycle that are not currently reached. New tools could potentially be developed to target outdoor blood-meal or sugar-meal feeding, for example, or to target mate-seeking or ovipositing females. Understanding the biology of these behaviours in the life cycle of important vectors could be the source of powerful new interventions. Even control approaches that achieve only a reduction in vector population density, such as interventions targeted at larvae, could prove valuable if they are sufficiently cost-effective and are complementary to existing tools.

Push-pull (repellent-attractant) technologies are well developed for some insect pests in the agricultural arena, but this technology has yet to be brought to bear on malaria control [26]. Our rapidly developing understanding of the mosquito sensory system, coupled to development of high-throughput screening technologies, should allow us to develop more effective attractants and repellents for mosquitoes within the next decade [27]. Modeling and experimental analysis of the impact of these compounds should allow us to develop new, targeted strategies for control. This technology also lends itself well to the extensive consumer market, which will undoubtedly play a major role in sustaining elimination efforts by reducing mosquito biting as mainstream vector control activities are reduced. Indeed, this is a situation that already exists in countries such as Sri Lanka and Mexico where the consumer market for products that reduce biting nuisance is high and national malaria control program vector control activities are minimal.

### Longer-Term Development of Novel Time-Limited Interventions

The past decade has seen phenomenal advances in *Anopheles* genomics and proteomics [28]. These advances, coupled with the visionary but technically challenging development of mosquito transgenics and other genetic manipulation techniques, open up the possibility of developing novel technologies to suppress mosquito populations or to make parasite-refractory mosquitoes, and make mosquito-based transmission-blocking technologies possible (Table 2). Such innovative new technologies may be key tools in the eradication agenda in the highly malaria-endemic regions of the world, in particular, much of sub-Saharan Africa, because they can circumvent the extreme problems in control program application that will be posed by intractable logistical, technical, or political issues in many of these regions. Importantly in intractable settings—for example, dense forests or politically unstable areas—where the elimination of malaria may prove most difficult, these technologies have the advantage that the mosquito population itself acts as the distribution agent.

Fortunately, the number of different vector species for which such technologies will need to be considered is limited, probably to only a handful of species. Moreover, the highly technical research needed to develop such tools for one major vector species will likely be fairly easy to adapt or even transfer directly to others. We now need to progress to the exacting but exciting translational phase of this activity, which will involve selection of the most appropriate technically robust technologies for operational implementation. Development, analysis, and refinement of scale-up technology to move progressively from the laboratory, to cage trials, and ultimately to operational scale release of genetically modified mosquitoes, and the establishment of the regulatory pathways for commercialization and release are all needed. Finally and critically, stakeholders, particularly in disease endemic countries, must be persuaded to support the release of genetically modified mosquitoes.

## Enabling Technologies

In order to establish TPPs for novel vector control products, particularly for products that target outdoor feeding or resting mosquitoes, we need to establish the critical points in the life cycle of these mosquitoes where they congregate in numbers, are susceptible to attraction by external stimuli, or come into contact with their human hosts. Better sampling methods that continuously track mosquito movement in space and time, rather than current methods that sample at known fixed points of interaction, are therefore needed. Moreover, methods that generate representative samples of mosquitoes in areas with intensive vector control activity are needed for accurate monitoring and evaluation of insecticide resistance and infection rates.

Cross-disciplinary initiatives are also needed to achieve a defined research agenda for improving engagement and communication with communities and all other stakeholders in malaria elimination. Such an agenda is needed to avoid the mistakes of past efforts, which have all too often foundered because of community fatigue after long years of engagement. Better integration of epidemiological expertise into vector control evaluation initiatives is also needed to ensure accurate field evaluation in increasingly complex multi-initiative settings, and a more commercially oriented approach to the development and evaluation of vector control technologies is required to ensure that promising initiatives cross the translational gap to implementation and poor technologies are rapidly discarded. Finally, cross-disciplinary initiatives are needed to achieve the rapid definition of efficient regulatory pathways and frameworks for existing and new technologies.

## Conclusions

On the basis of its deliberations, the malERA Consultative Group on Vector Controls proposes a research and development agenda for vector control (Box 1). The first of these agenda items—the development of an analytical framework to facilitate decision making—is achievable in the next 12–18 months. The other areas need to be rapidly progressed over the next decade. It will be up to everyone involved in malaria elimination/eradication to work together to ensure that all the needs and goals highlighted in this agenda are achieved as efficiently as possible. Importantly, however, our proposed agenda provides a starting point only for the research and development needs associated with vector control. This agenda must not be set in stone; it must continue to evolve as the elimination/eradication program progresses.

**Box 1. Summary of the Research and Development Agenda for Vector Control**

- Development of an analytic framework that can bring together existing and new information on all aspects of malaria and malaria transmission through a public portal designed to facilitate decision making by the malaria research, control, and tool development communities.
- An improved choice of insecticides, and formulations coupled with improved methods to reduce the risk of resistance to ensure that the availability of effective insecticides does not become the limiting factor in our ability to reduce transmission to levels where local elimination can be attempted.
- Better understanding of the ecology, behaviour, and genetic population structure of malaria vectors, particularly outdoor biting and resting species that escape current vector control tools.
- Development of innovative new technologies that can:
  - Educate the community effectively and engage the consumer market
  - Control outdoor biting and resting mosquito vectors
  - Simply and rapidly measure transmission
- Sustained commitment to the long-term development of novel approaches like the genetic manipulation of natural vector populations that will permanently reduce the very high vectorial capacities of dominant malaria vectors in sub-Saharan Africa and some parts of Asia.

## Abbreviations

GMEP: Global Malaria Eradication Program
IRS: indoor residual insecticide spray
LLIN: long-lasting insecticide-treated net
TPP: target product profile
